# Assessment of body fat percentage in Emirati females: a comparative analysis of BIA vs. DXA

**DOI:** 10.3389/fnut.2025.1717492

**Published:** 2026-01-09

**Authors:** Dalia Haroun, Aseel Ehsanallah

**Affiliations:** Department of Health Sciences, College of Natural and Health Sciences, Zayed University, Dubai, United Arab Emirates

**Keywords:** BIA, DXA, Emirati, females, body composition

## Abstract

**Introduction:**

Obesity is a significant health issue in the UAE. Accurate body composition assessment is crucial for managing obesity-related health risks. This study aimed to evaluate the agreement between bioelectrical impedance analysis (BIA) and dual-energy X-ray absorptiometry (DXA) in measuring body composition among Emirati females.

**Methods:**

This cross-sectional study involved 95 healthy Emirati females aged 17–27 years. Paired samples *t*-tests, correlation analyses, and Bland-Altman plots were used to compare the two methods (BIA vs. DXA).

**Results:**

BIA significantly underestimated % fat and fat mass (FM) while overestimating fat-free mass (FFM) compared to DXA. The mean difference in % fat was −14.1% (*p* < 0.001), and the mean difference in FFM was +8.2 kg (*p* < 0.001). Despite strong correlations between BIA and DXA measurements (*r* = 0.855 for % fat, *r* = 0.984 for FM, and *r* = 0.929 for FFM), Bland-Altman plots indicated poor agreement, with wide limits of agreement.

**Conclusion:**

Bioelectrical impedance analysis remains valuable for obesity assessment in large-scale studies and clinical settings due to its non-invasive, easy-to-use, and cost-effective characteristics. The results show that the in-built prediction equations cannot adequately predict the % fat, FM, and FFM for this sample. Future research should focus on developing and validating BIA-specific equations tailored for Emiratis.

## 1 Introduction

Obesity has emerged as a pressing health concern for the United Arab Emirates (UAE) in recent years. Data extracted from the UAE National Health Survey Report of 2017–2018 reveals a high prevalence of obesity among adults, estimated at around 27.8% (1). This statistic indicates a broader health context dominated by non-communicable diseases (NCDs), which the World Health Organization (WHO) identifies as the primary cause of mortality in the region. In fact, NCDs account for 55% of all deaths in the UAE, with cardiovascular diseases (CVDs) emerging as the primary culprit, responsible for 34% of these fatalities (2). Therefore, addressing the increasing prevalence of obesity and its related health risks has become an urgent priority in the UAE.

Assessing obesity requires methods that are not only accurate but also cost-effective and efficient for use in large-scale settings. Weight and height measurements offer a simpler and quicker alternative, with body mass index (BMI) being a widely employed metric (3). BMI’s limitations include its inability to differentiate between muscle, fat, bone, or vital organs, leading to misclassification of individuals with high fat-free mass (FFM) relative to stature as overweight or obese, and its failure to account for variations in body composition among individuals (4, 5). Moreover, body fat percentage (% fat) varies with age, sex, ethnicity, and individual differences, complicating the interpretation of BMI values (6). Although BMI has its limitations, it remains popular in epidemiological research. Alternative methods for assessing obesity, such as body fat-mass measurement techniques including bioelectrical impedance analysis (BIA), Computed Tomography (CT), Magnetic Resonance Imaging (MRI), and dual-energy X-ray absorptiometry (DXA), are considered more precise for the assessment of body composition.

Dual-energy X-ray absorptiometry is renowned as the gold standard for bone mineral content assessment and is recognized for its precision and applicability in various settings (7–9). Unlike conventional X-ray systems, DXA requires special beam filtering and precise spatial registration to measure both whole-body bone mass and soft tissue composition (10). DXA utilizes a 3-compartment model, with compartments encompassing fat mass (FM), lean and soft tissue mass (LSTM), and bone mineral content. Increasing the number of compartments enhances accuracy, lowers the chances of measurement errors, and reduces the need for assumptions in determining body composition (11). DXA assessments are rapid, non-invasive, and entail minimal inconvenience for patients, further enhancing its appeal in clinical practice and research (12). However, despite its advantages, DXA does have drawbacks, including the requirement for expensive specialized radiology equipment, small radiation exposure, and the need for trained technicians, which may limit its feasibility in routine clinical practice (12, 13).

To address these challenges, BIA has been suggested to assess body composition. BIA is used to assess body composition, relying on parameters such as impedance and phase angle to estimate different body compartments (14). It utilizes the body’s electrical properties to gauge resistance to an electric current. The measured impedance, along with factors such as weight, height, and age, is used in prediction equations to first estimate FFM or total body water. A total of % fat and FM are then typically derived from these estimates (6, 15–18). BIA operates under the assumption of constant FFM density. However, significant water fluctuations during growth and development can lead to inaccuracies in body composition measurements (13). Other factors, including device type, water distribution, hydration status, weight, and height, may increase the risk of inaccurately assessing FFM (6, 19). BIA is safe, simple, non-invasive, and cost-effective, making it increasingly popular for evaluating body composition in clinical and research settings (12, 13, 20, 21). It has emerged as a popular alternative to DXA for assessing body composition (9). BIA’s advantages, such as portability, affordability, minimal training requirements, and lack of radiation exposure, make it a practical option for assessing body composition in both clinical practice and large-scale epidemiological studies (15, 19, 22–24). However, the accuracy of predictive equations for estimating TBW depends on factors such as the type of BIA device used to record impedance data, the reference method employed to assess FFM, and the characteristics of the population in which the equation was developed (25).

Bioelectrical impedance analysis devices incorporate predictive equations for body fat that were originally developed using data from specific demographic groups (4, 12). Many of these equations were initially derived from studies involving predominantly white populations and subsequently applied to individuals from diverse ethnic backgrounds. However, research indicates that there are variations in body composition among different ethnic groups, potentially impacting the precision of BIA measurements (26–29). Hence, past research suggests the necessity for customized BIA equations tailored to specific ethnic groups (30, 31). Currently, there is a lack of research comparing BIA and DXA in the UAE. Therefore, the aim of this study was to examine the agreement between BIA and DXA measurements of body composition among Emiratis.

## 2 Materials and methods

### 2.1 Study subjects

In a retrospective analytical cross-sectional study, data, including questionnaire responses and measurements, were collected initially between March 2016 and March 2017. This study involved analyzing a comprehensive dataset obtained from a sample of 170 females aged 17 to 27 years. The participants were healthy Emirati women living in the UAE, selected using a convenience snowball sampling method. Participants were selected based on specific inclusion criteria, including female Emiratis aged between 17 and 28, having fasted for 12 h, and not currently menstruating. Exclusion criteria encompassed non-Emiratis, individuals with metabolic disorders (such as diabetes, kidney disease, and hypertension), those taking certain medications, pregnant or lactating females, and those reporting weight fluctuations exceeding 3 kg. The exclusion of menstruating participants was necessary due to the potential for fluid retention and shifts in body water distribution during the menstrual cycle (32). Although recent evidence suggests BIA measurements are not significantly affected by the menstrual cycle (32–34), this exclusion was implemented as a conservative measure. Participants who met the criteria were provided with an information sheet detailing the preparation requirements. To ensure adherence, they received a reminder phone call the day before their appointment, and compliance was confirmed upon arrival. Following the application of these criteria, 95 out of the initial 170 participants were included in the analysis. A minimum sample of 91 participants was determined (power = 0.8, alpha = 0.05) to achieve a medium effect size for the coefficient of determination (R^2^) increases in a regression equation with up to five predictors. Therefore, our sample of 95 participants was enough to ensure adequate power analysis in the equation development (35).

Participation in the study was voluntary and informed written consent was obtained from all participants. Ethical approval for the study was obtained from the Zayed University Research Ethics Committee (ZU16_016_F). Participants who agreed to take part were provided with a consent form to review and sign before the assessment session. To maintain confidentiality, the names and contact details of the participants were kept separately in a secure cabinet, and each participant was assigned a unique code. Various communication channels, such as email, text messages, and campus word of mouth, were utilized to disseminate information about the study and recruit female university students. Interested females were provided with either an electronic or paper copy of the information sheet upon expressing their willingness to participate.

Measurements were conducted in the body composition laboratory at Zayed University in Dubai, UAE. All participants needed to arrive at the laboratory after fasting for at least 8–12 h and with an empty stomach. All the measurements were taken on the same day. Participants were instructed to wear light clothing, avoid any physical activity, refrain from consuming caffeinated beverages, and empty their bladders before measurements were taken. A checklist was used to ensure that the participants met the eligibility criteria for the examination. The session, which lasted between 60 and 90 min, involved the collection of data and measurements.

### 2.2 Data collection

#### 2.2.1 Questionnaire

A trained research assistant administered a structured questionnaire to gather personal information, including age, sex, ethnicity, marital status, medical history, and any medications used, if relevant. Participants were also asked about weight fluctuations over the past 3 months, with the question: “Have you experienced any changes in your weight? If so, How many kilograms?”

#### 2.2.2 Anthropometric measurements and body fat analysis

Body weight was measured to the nearest 0.1 kg and height was measured to the nearest 0.1 cm using a wall-mounted stadiometer. BMI was calculated by dividing the weight in kilograms by the square of the height in meters (kg/m^2^). WHO defines overweight as a BMI between 25 and 29.9 kg/m^2^, and the classification of obesity is defined as a BMI equal to or greater than 30 kg/m^2^ (36).

#### 2.2.3 Dual energy X-ray absorptiometry

The DXA scanner provides accurate data on bone and soft tissue composition, including bone mineral density (BMD), lean and fat tissue mass, and fat percentage. The DXA scanner (Lunar iDXA, GE Healthcare) was calibrated daily according to the manufacturer’s instructions using a proprietary quality assurance phantom to ensure measurement precision. Participants were positioned supine on the scanning table, wearing light, metal-free clothing. Their hands were placed at their sides, and to refrain from moving throughout the measurement. The whole-body scan mode was used for all participants, and scans were analyzed using the manufacturer’s enCORE software.

#### 2.2.4 Bioelectrical impedance analysis

Bioelectrical impedance analysis was performed using a TANITA BC-418 MA segmental body composition analyzer (TANITA UK LTD). This device operates at a single frequency of 50 kHz and uses eight tactile electrodes: two for each hand and two for each foot. Prior to measurement, participants’ age, sex, height, and standard body type were entered into the system. Inaccuracies in these inputs affect the body composition estimates but not the resistance measurement. Participants stood barefoot on the device’s footplate electrodes and gripped the hand-held electrodes, keeping their arms slightly away from their torso for approximately 1 min. The BIA printout includes an estimate of % fat, FM, FFM, and measured whole-body resistance/impedance (Ω).

### 2.3 Statistical analysis

Statistical analyses were performed using the Statistical Package for Social Sciences software (SPSS version 29). Descriptive statistics, including means and standard deviations, were calculated for participant characteristics. Paired samples *t*-tests were used to assess if measures of % fat, FM, and FFM were significantly different between BIA and DXA. The level of statistical significance was set at *p* < 0.05. Cohen’s d for paired samples was calculated as a measure of effect size, where *d* = 0.2 was considered a small effect, *d* = 0.5 a moderate effect, and *d* = 0.8 a large effect (37). Pearson correlation coefficients were used to assess the correlation between % fat, FM, and FFM measured by BIA and DXA. Agreement between both the standard and new BIA equations with DXA was assessed using Bland-Altman analysis, which was used to determine the mean bias, 95% limits of agreement, and the presence of proportional bias for each method (38). For the development of a population-specific equation, FFM obtained by DXA was used as the dependent variable. A stepwise linear regression was first performed with resistance index (RI = height^2^/resistance), body mass, and age as independent variables to identify the most relevant predictors. The model’s residuals were tested for normality and homoscedasticity. During model development, the following criteria and diagnostics were used: the coefficient of determination (R^2^) and standard error of the estimate (SEE) were reported to evaluate the model’s fit and predictive accuracy. Multicollinearity was assessed using the variance inflation factor (VIF) and tolerance, with a VIF < 10 and tolerance > 0.1 considered acceptable. However, acknowledging the inherent non-linear relationship between conductive volume and bioelectrical resistance, a non-linear regression model was subsequently applied to develop the final prediction equation. The validity of the final proposed equation was verified by comparing its estimates against DXA using a paired samples *t*-test, Pearson’s correlation coefficient, the intraclass correlation coefficient (ICC), SEE, and pure error (PE).

## 3 Results

The descriptive characteristics of all 95 participants included in the analysis are shown in Table 1. The average age of the subjects was 19.7 ± 2.0 years, and the mean BMI was 22.9 ± 5.2 kg/m^2^. Out of the 95 participants, 19 (20%) were classified as underweight, 52 (54.7%) had a normal weight, 14 (14.7%) were overweight, and 10 (10.5%) of the participants had obesity.

**TABLE 1:** Descriptive characteristics of the study sample (*n* = 95).

Variable	Minimum	Maximum	Mean ± SD
Age (years)	17	28	19.7 ± 2
Height (cm)	139.5	180	158.4 ± 6.4
Weight (kg)	36.1	113.8	58 ± 15.4
BMI (kg/m^2^)	14.8	38.9	22.9 ± 5.2
Waist circumference (cm)	52	105	68.7 ± 10.8
Tanita % fat	3	50.6	24.9 ± 10.2
Tanita FM (kg)	1.1	57.6	15.9 ± 10.8
Tanita FFM (kg)	34.6	61.6	42.1 ± 4.9
Resistance (Ω)	432.7	920.8	622.9 ± 93.1
Resistance index (cm^2^/Ω)	27.7	74.9	41.4 ± 7.9
DXA % fat	23.9	56.2	39.1 ± 6.8
DXA FM (kg)	8.9	57.6	22.1 ± 10.2
DXA FFM (kg)	22.1	57.5	33.9 ± 6.3

BMI, body mass index; FM, fat mass; FFM; fat-free mass.

A paired samples *t*-test was conducted to assess differences in % fat, FM, and FFM between BIA and DXA (Table 2). Results indicate that BIA significantly underestimated % fat (14.1%) and FM (5.8 kg) compared to DXA, with a large effect size and a strong correlation between the two methods. Conversely, BIA significantly overestimated FFM by 8.2 kg compared to DXA, also showing a large effect size and strong correlation. All three body composition parameters measured by DXA and BIA showed significant positive correlations (*p* < 0.01).

**TABLE 2:** Stepwise linear regression analysis of predictors influencing fat-free mass (FFM) measured by dual-energy X-ray absorptiometry (DXA).

Model	Variable	R^2^	SEE	*P*-value	Tolerance	VIF
1	Resistance index	0.803	2.81	<0.001	1.0	1.0
2	Resistance index	0.877	2.23	<0.001	0.293	0.293
	Body mass			<0.001	0.293	0.293
3	Resistance index	0.906	1.96	<0.001	0.292	3.420
	Body mass			<0.001	0.292	3.423
	Age			<0.001	0.996	1.004

**FIGURE 1:**
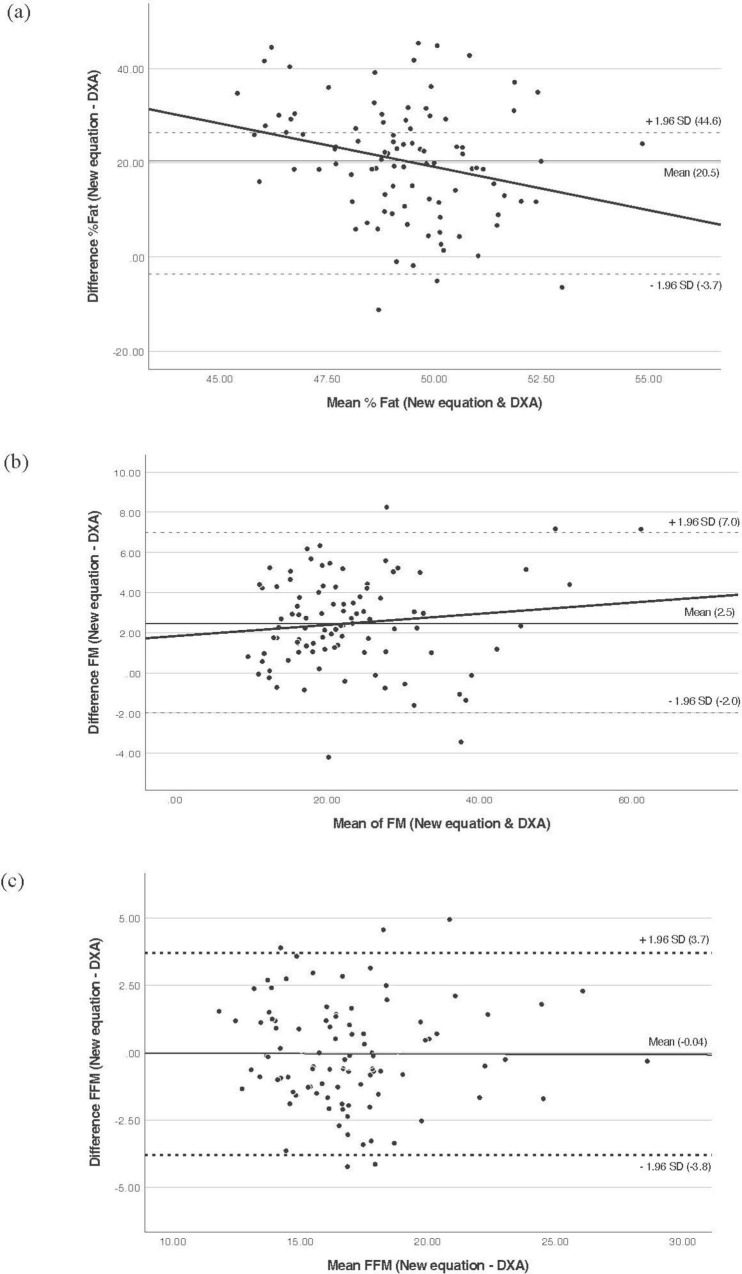
Bland and Altman plot analysis to evaluate the agreement between BIA and DXA. **(a)** % Fat, **(b)** FM, **(c)** FFM. The middle solid line represents fixed bias (mean difference), the dashed lines represent 95% limits of agreement, and sloped trend line demonstrates proportional bias. BIA, bioelectrical impedance analysis; DXA, dual-energy X-ray absorptiometry; FFM, fat-free mass; FM, fat mass; SD, standard deviation.

The Bland–Altman plots (B&A) were used to determine the LoA between BIA and DXA (Figure 1). The B&A plot for % fat and FM indicate that BIA tends to underestimate % fat to a greater extent among those with greater % fat compared to those with lower % fat (LoA: −25.1 to −3.1). A similar observation was seen for FM, where the discrepancy between BIA and DXA increases with higher FM, indicating proportional bias for both % fat and FM. For FM (LoA: −10.0 to −1.6). For FFM, the non-zero slope of the trend line also indicates a proportional bias. The mean difference in FFM estimates was larger for individuals with lower FFM, with BIA overestimating FFM in participants with lower FFM compared to those with higher FFM (LoA: 3.3–13.2). Across all measures, the greatest overestimate was 13.37 for FFM, and the greatest underestimate was −27.5 for %fat. These results show that the in-built prediction equations cannot adequately predict the %fat, FM, and FFM for this sample.

Following the assessment of the standard BIA’s performance, a stepwise linear regression was conducted to develop a population-specific equation (Table 3). The regression identified RI, body mass, and age as significant predictors of FFM. The final linear model yielded an R^2^ of 0.906 and an SEE of 1.96 kg. Collinearity diagnostics confirmed the absence of multicollinearity, with tolerance values of 0.292 for RI and body mass (VIF = 3.4) and a tolerance of 0.996 for age (VIF = 1.0).

**TABLE 3:** Paired samples *t*-test results comparing % fat, fat mass (FM), and fat-free mass (FFM) from the built-in and proposed equations against dual-energy X-ray absorptiometry (DXA).

Variable	Mean difference	SD	*P*-value	Cohen’s d	Correlation	PE	SEE	ICC
**Built-in equation**								
% fat	−14.1	5.6	<0.001	−2.5	0.855*	15.1	3.54	0.842
FM (kg)	−5.8	2.2	<0.001	−2.7	0.984*	6.2	1.73	0.341
FFM (kg)	8.2	2.5	<0.001	3.2	0.929*	8.6	2.34	0.435
**New equation**								
% fat	20.5	12.3	<0.001	1.7	−0.857*	23.8	3.53	−0.139
FM (kg)	2.5	2.3	<0.001	1.1	0.973*	3.35	2.22	0.943
FFM (kg)	−0.04	1.9	0.833	−0.02	0.952*	1.92	1.94	0.951

FM, fat mass; FFM; fat-free mass; ICC, intraclass correlation coefficient; PE, pure error; SEE, standard error of estimate. Mean difference, BIA−DXA. *Correlation is significant at the 0.001 level.

The subsequent non-linear regression produced the final prediction equation, which retained RI (β = 0.396), body mass (β = 0.198), and age (β = 0.537) as significant predictors. The resulting Emirati-specific equation for FFM:


F⁢F⁢M=-4.599+0.396⁢(R⁢I)+ 0.198⁢(b⁢o⁢d⁢y⁢m⁢a⁢s⁢s)+0.537⁢(a⁢g⁢e)

**FIGURE 2:**
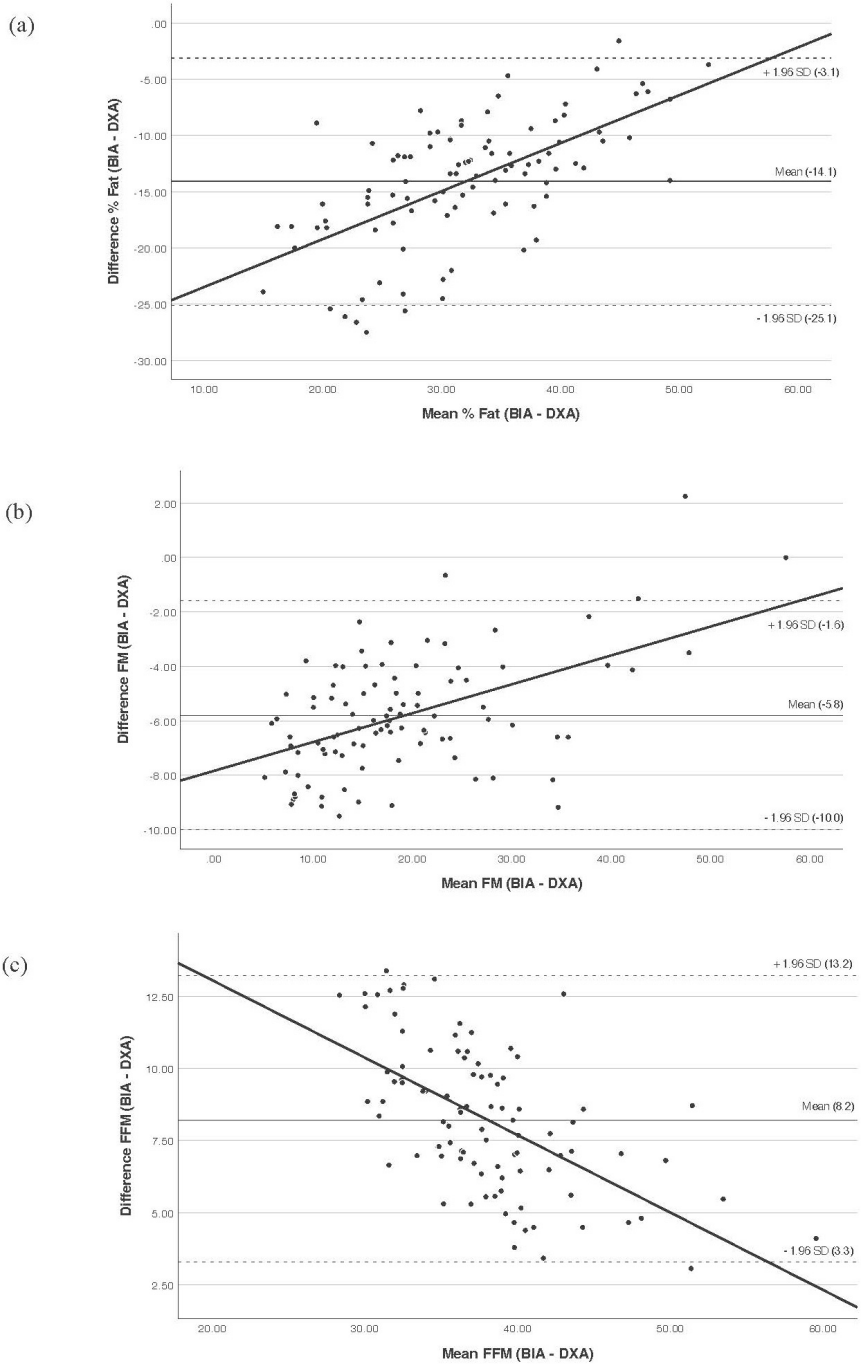
Bland and Altman plot analysis to evaluate the agreement between the developed population-specific equation and DXA. **(a)** % Fat, **(b)** FM, **(c)** FFM. The middle solid line represents fixed bias (mean difference), the dashed lines represent 95% limits of agreement, and sloped trend line demonstrates proportional bias. BIA, bioelectrical impedance analysis; DXA, dual-energy X-ray absorptiometry; FFM, fat-free mass; FM, fat mass; SD, standard deviation.

The performance of this new equation was then evaluated against DXA (Table 3). The results demonstrate that the new equation successfully eliminated the systematic error in estimating FFM, reducing the bias from +8.2 to −0.04 kg and achieving excellent agreement (ICC = 0.951). It also substantially improved the estimation of FM (ICC = 0.943). B&A plots (Figure 2) confirmed these findings, showing a negligible mean bias for FFM with narrow 95% limits of agreement (LoA: −3.8 to +3.7 kg). For FM, the new equation showed a reduced mean bias of +2.5 kg (LoA: −2.0 to +7.0 kg) and no proportional bias. For % fat, the new equation introduced a systematic overestimation, in contrast to the underestimation seen with the standard equation.

## 4 Discussion

This study evaluated the agreement between BIA and DXA for measuring body composition in a sample of 95 Emirati females. Our findings demonstrate that, despite strong correlations, the in-built BIA prediction equations showed poor agreement with the reference DXA method and are not adequate for this demographic group. Specifically, BIA significantly underestimated % fat and FM by 14.1% and 5.8 kg, respectively, while overestimating FFM by 8.2 kg. This pattern of systematic bias, which could lead to misclassification at the individual level (e.g., % fat differences ranging from −3.1% to −25.1%). Although there was a strong correlation overall, the agreement at the individual level was not clinically acceptable.

This aligns with findings from validation studies in other populations. A study by Lopes et al. in a sample of 121 adults observed a strong positive correlation between BIA and DXA in measuring FFM and % fat, yet emphasized that these methods are not interchangeable. BIA consistently underestimated % fat by 5.56% and overestimated FFM by 2.90 kg, which could potentially impact nutritional planning by clinical dietitians and patient outcomes (39). Leahy et al.’s study, conducted on a sample of 403 healthy young Irish adults, similarly found that BIA consistently underestimated both % fat and FM compared to DXA, with a more pronounced effect in women and individuals with higher total % fat (40). Another study conducted on Turkish students reported comparable findings that BIA consistently underestimated % fat compared to DXA, with the degree of underestimation remaining consistent across varying levels of body fat (41). With numerous studies suggesting that BIA tends to underestimate % fat, particularly in individuals with higher BMI, its utility remains viable as a practical option for body composition assessment in the absence of more precise methods (27). At the segmental level, Baglietto et al. found in a study aimed at comparing body composition methods that DXA and BIA are not interchangeable for assessing segmental weight, FFM, or LSM. Using DXA as a reference, BIA consistently overestimated segmental weight in the trunk and upper limbs and underestimated it in the lower limbs (42).

Given the systematic bias observed with the manufacturer’s equation, this study developed a novel, population-specific prediction equation for FFM using non-linear regression. The performance of this new equation represents a substantial improvement over the standard BIA equation. Most notably, for FFM, the new equation successfully eliminated the large systematic bias, reducing the mean difference from +8.2 to −0.04 kg, and achieved excellent agreement with DXA (ICC = 0.951). The LoA were also narrowed, enhancing its reliability for individual-level assessment. Similarly, for FM, the new equation showed a greatly reduced bias and excellent agreement (ICC = 0.943).

Our study adds to a growing body of literature demonstrating the necessity of developing population-specific BIA equations. The suboptimal performance of general equations in our sample is consistent with findings from other ethnic groups. This is further reinforced by the recent work of Masset et al. who demonstrated that established BIA equations were invalid for Brazilian adults with overweight and obesity. They developed and cross-validated new population-specific equations that met all validity criteria against DXA. Their findings highlight a critical gap in standard practice, noting that while using population-specific equations is crucial to reduce discrepancies (43). Similarly, Costa et al. developed and cross-validated new BIA equations for Brazilian women, finding that previously published equations from other populations were not valid for their sample. The newly developed equations showed excellent validity against DXA. The study concluded that the best solution would be for manufacturers to provide different predictive equations based on subject characteristics, and that if equipment lacks this feature, professionals should use raw impedance data with a theoretically valid equation (16). The need for population-specific equations extends even to Caucasian populations. Rojano-Ortega et al. developed and validated new equations for a heterogeneous Caucasian cohort in Spain for both hand-to-hand and foot-to-hand devices. Their new equations showed higher coefficients of determination, lower SEE, and ICCs than the manufacturers’ equations, demonstrating significantly improved accuracy (44).

Variations in BIA results are influenced by differences in device parameters, including the path of the electrical current, electrode configuration, participant positioning, frequency settings, and specific equations used (13). Another factor contributing to variability in BIA measurements is its susceptibility to bias in populations with altered hydration levels, such as during fasting, pregnancy, medication intake, and obesity. These biases arise primarily due to assumptions of consistent properties in FFM. However, the use of age, sex, and population-specific equations can enhance the accuracy of BIA in estimating body composition (27). One study reported a strong agreement between BIA and other methods for measuring FFM and FM but noted that BIA equations are specific to manufacturers and populations, often derived from data of healthy individuals with normal weight (14). These assumptions are critical for accurate BIA outcomes, and deviations can impact measurement precision. Developing tailored prediction equations for specific ethnic populations may further improve agreement between BIA and DXA (23).

The primary reason for these ethnic-specific equations in BIA is likely due to recognized differences in physique and body proportions between ethnic groups (29). These differences include variations in stature and lean mass. Moreover, there are notable differences in the amount and distribution of body fat across ethnicities (31). Because impedance measurements are influenced by the body’s cross-sectional area and the length of conducting segments, ethnic differences in body size and proportions may contribute to the ethnic-specific relationship observed between bioelectrical data and body composition (29).

The increasing prevalence of obesity in the UAE highlights the urgent need for precise methods to assess body composition. Accurate assessment methods are vital for developing effective public health interventions and clinical strategies to combat obesity and its associated health risks. From a public health perspective, reliance on body composition tools that systematically misestimate FM or lean mass may contribute to incorrect energy and macronutrient targets. In populations with high rates of overweight and obesity, even small inaccuracies can have meaningful consequences. Overestimation of lean mass or underestimation of FM may lead to inflated energy requirement estimates, which can inadvertently promote positive energy balance and hinder weight-management efforts. Given the well-established association between excess energy intake and obesity prevalence, ensuring accurate body composition assessment is critical to designing effective interventions, monitoring progress, and guiding individualized clinical care. Thus, validating BIA against a reference method such as DXA is not only methodologically important, but also essential for translating body composition data into accurate, culturally relevant, and population-specific nutrition guidance and public health strategies.

Although numerous studies have proposed new predictive equations to enhance the accuracy of body composition assessments using BIA, no studies have been conducted in the UAE. This study, therefore, addresses a critical methodological gap regarding BIA accuracy in Middle Eastern females and represents a first attempt to generate a population-specific BIA prediction equation for this demographic, providing a foundational tool for more accurate body composition assessment. While our study provides valuable insights into the agreement between BIA and DXA measurements of body composition in Emirati females, several limitations should be acknowledged. The study’s reliance on convenience sampling may introduce selection bias, potentially limiting the generalizability of the findings to the broader population of Emirati females. The small sample size restricted our ability to compare the agreement between BIA and DXA across different BMI categories and hindered the capacity to validate the proposed equation in an independent sample. Additionally, hydration status was not assessed, which may have influenced the accuracy of BIA measurements. Furthermore, this approach should not be used to track changes in FFM, as the equation is derived from cross-sectional data rather than longitudinal analysis.

In conclusion, this study identified substantial discrepancies between standard BIA equations and DXA in Emirati females. BIA consistently underestimated % fat and FM while overestimating FFM. Despite these discrepancies, BIA remains a valuable tool for obesity assessment due to its ease of use, non-invasiveness, and cost-effectiveness. Our study developed a new Emirati-specific equation that, in our sample, successfully eliminated the systematic bias for FFM and substantially improved the estimation of FM. While these initial results are highly promising, this preliminary equation requires external validation in an independent sample and is currently applicable only to females. Therefore, future research should focus on validating BIA-specific equations tailored for the Emirati population to enhance their accuracy and reliability.

## Data Availability

The original contributions presented in this study are included in this article/supplementary material, further inquiries can be directed to the corresponding author.
